# Anterior Crossbite Correction in Early Mixed Dentition Period Using Catlan's Appliance: A Case Report

**DOI:** 10.5402/2011/298931

**Published:** 2010-11-23

**Authors:** Prashanth Prakash, B. H. Durgesh

**Affiliations:** ^1^Department of Pediatric Dentistry, Manubhai Patel Dental College and Charitable Hospital, Vishwajyoti Ashram, Munjmahuda, Vadodara - 390011, Gujarat, India; ^2^Department of Orthodontics, Mauras College of Dentistry and Hospital and Oral Research Institute, Arsenal, Mauritius

## Abstract

Single tooth anterior dental crossbite is the commonly encountered malocclusion during the development of occlusion in children. Various treatment options such as removable and fixed appliances have been suggested by different authors in the past literature. This paper presents two cases of anterior crossbite corrected using the standard Catlan's appliance (Lower Inclined Bite Plane) in a short period of three weeks without any damage to the tooth or the periodontium. This fixed appliance is a simple and traditional method which does not depend on patient cooperation to reverse the bite.

## 1. Introduction

Anterior crossbite is a major esthetic and functional concern to the parents during the developmental stage of a child. It is one of the major responsibilities of pediatric dentist or orthodontist to guide the developing dentition to a state of normalcy in line with the stage of oral-facial growth and development [1]. The period of mixed dentition offers the greatest opportunity for occlusal guidance and interception of malocclusion [2]. If delayed to a later stage of maturity, treatment may become more complicated [3]. Also, there is relatively very few documented case reports about the use of Catlan's appliance in the treatment of anterior crossbite. Henceforth, this paper documents two cases in which anterior dental crossbite were successfully corrected using a simple fixed Catlan's appliance. 

Graber has defined crossbite as a condition where one or more teeth may be abnormally malposed either lingually or labially with reference to opposing teeth [4]. Anterior crossbite is defined as a malocclusion resulting from the lingual positioning of the maxillary anterior teeth in relationship to the mandibular anterior teeth [5]. Anterior crossbite is also defined as upper frontal primary or individual permanent teeth lingual position in relationship to the lower incisor teeth [6].

Severe anterior crossbite in contrast to posterior crossbite are usually not corrected until the second stage of conventional treatment or might remain pending for surgical correction. The early mixed dentition stage provides an ideal platform to use this Catlan's appliance and reverse the bite. To use this appliance, the practitioner has to first distinguish crossbite of dental origin from those of skeletal origin [4, 7–9]. Dental crossbite involves localized tipping of a tooth or teeth and does not involve basal bone [10]. In the simple anterior dental crossbite, the patient should display a normal skeletal pattern with abnormalities presenting in the axial inclination of the affected teeth only [8]. According to Profitt, correction of anterior dental crossbite requires first opening of enough space, then bringing the displaced tooth or teeth across the occlusion into proper position [11]. 

Anterior dental crossbite has a reported incidence of 4-5% and usually becomes evident during the early mixed-dentition phase [12, 13]. The anterior crossbite may result from variety of factors such as lingual eruption path of the maxillary anterior incisors; a repaired cleft lip; trauma to the primary incisor resulting in lingual displacement of the permanent tooth germ; supernumerary anterior teeth; an over-retained necrotic or pulpless deciduous tooth or root; odontomas; crowding in the incisor region; inadequate arch length; a habit of biting the upper lip [9, 10, 12–15].

Anterior crossbite may lead to abnormal enamel abrasion of the lower incisors, dental compensation of mandibular incisors leading to thinning of labial alveolar plate, and/or gingival recession [4, 7–9]. Anterior dental crossbite requires early and immediate treatment to prevent anterior teeth mobility and fracture, periodontal pathosis, and temporomandibular joint disturbance [7, 9, 15, 16].

The main goal of treatment is to tip the affected maxillary tooth or teeth labially to the point where a stable overbite relationship exists [16]. Relapse is usually prevented by the normal overjet/overbite relationship that is achieved [17]. Treatment modalities for correction of anterior crossbite are tongue blades, reversed stainless steel crowns, fixed acrylic inclined planes, bonded resin-composite slopes, removable acrylic appliances with finger springs, and Bruckl appliance [9, 10, 14].

## 2. Case Reports

**Case 1**

**Figure 1:**
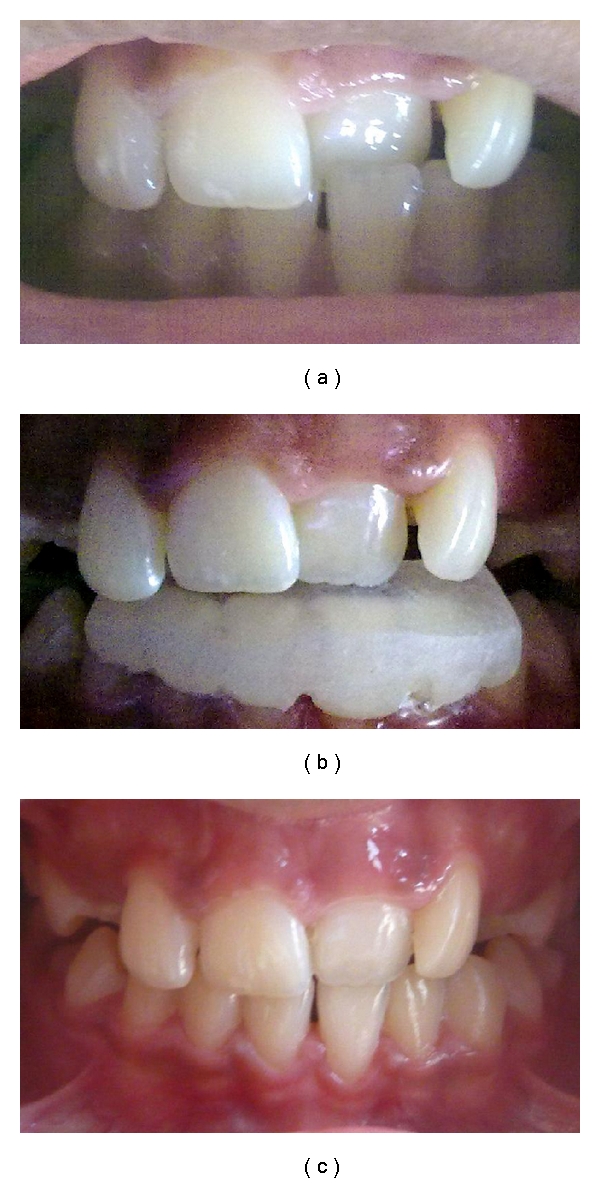
(a) A 9-year-old girl showing anterior dental crossbite. (b) Catlan's appliance (Lower Inclined Bite Plane) cemented. (c) Posttreatment incisor relation after 3 weeks.

A 9-year-old female patient accompanied by her parents reported to the hospital with a chief complaint of sensitivity in the upper right and left back teeth region since two days which aggravates on having food and relieved after few seconds. A complete clinical examination revealed the permanent maxillary left central incisor in crossbite (Figure 1(a)) along with dental caries in 16, 14, 26, 36, and 46. Following clinical and radiographic examinations, the decision was made to fabricate an inclined plane. The parents were informed about the malocclusion, and a written consent to proceed with the treatment was taken. The crossbite was corrected after the cementation of the Catlan's appliance within three weeks (Figure 1(c)). During the subsequent visit to the dentist, other restorative procedures were carried out. Recall examination after 6 months showed normal incisal relation without any relapse.

**Case 2**

**Figure 2:**
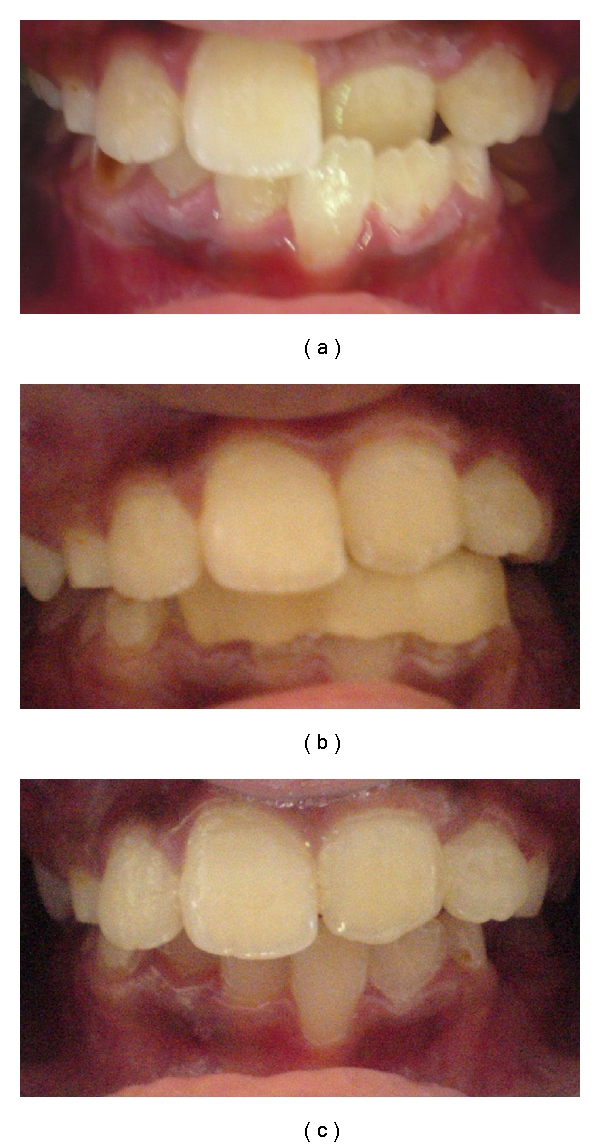
(a) A 9-year-old-boy with anterior dental crossbite. (b) Lower Inclined Bite Plane is cemented. (c) Frontal view showing correction of crossbite after 3 weeks.

A 9-year-old male patient was referred to the pediatric dental department with a chief complaint of broken milk teeth and esthetic concern of the front tooth. On clinical examination, anterior crossbite was observed in relation to maxillary left central incisors (Figure 2(a)) along with retained root stumps in the posterior region of the oral cavity. Parents were informed about the treatment, and a written consent was documented. The crossbite was treated with inclined plane within a span of three weeks, and the bite was reversed without any undue problems to the child (Figure 2(c)). The patient was examined after 7 months, and there was no relapse of the crossbite in relation to maxillary left incisor.

Both the cases reported here were in early mixed dentition and had class I molar and canine relationships. In every case, there was sufficient mesiodistal width to achieve labial movement of the maxillary tooth. Alginate impressions of both arches were taken, and an acrylic inclined plane with a slope of 45 degree angulations to the long axis of the tooth was established. The inclined plane was cemented on to the mandibular incisors and canines with zinc oxide eugenol cement (Figures 1(b) and 2(b)). After the cementation of the inclined plane, the only contact point was present at the incisor region in state of occlusion. The patients were advised to maintain good oral hygiene and recalled every week to clinically evaluate the progress of the treatment. The parents were told that the child's bite will feel unusual for a while, but the child will adjust to it and a softer diet than usual was suggested for the first few days after the cementation. Following correction, the Catlan's appliance was removed, the enamel surface was polished, and topical fluoride (APF gel) was applied. Recementation was not required in both cases due to the adequate retention of the appliance during the follow-up examinations.

## 3. Discussion

Anterior crossbite is a condition which seldom corrects by itself because the maxillary incisor is locked behind the mandibular incisors and continues to progress leading to severe malocclusion, thus early treatment can reestablish proper muscle balance and a well balanced occlusal development. Early treatment is also directed towards preventing dysplastic growth of both skeletal and the dentoalveolar components [18]. The Lower Inclined Bite Plane is the traditional method used for correcting anterior single tooth or multiple tooth dental crossbite. It has to be used only if there is enough space in dental arch for labial movement of the upper incisors. Clinically it can be used in cases when upper incisors are in crossbite with more than one half of vertical overbite. The movement of teeth occurs from the resulting force of closing muscle and inclined plane interaction. One of the shortcomings of early treatment is the possibility of a two-phase orthodontic therapy as often it is difficult to estimate the further growth of the mandible [19]. 

The case selection for using this appliance determines the success of the treatment as it depends on three basic factors given by Lee 1978 which include adequate space in the arch to reposition the tooth, sufficient overbite to hold the tooth in position following correction, and a class I molar relation [7]. The presence of crowding in mandibular incisors, tempromandibular joint problems, and maxillary deficiency has to be considered before suggesting this appliance. The ideal age for the correction of anterior dental crossbite is between 8 to 11 years during which the root is being formed and the tooth is in the active stage of eruption. The important role plays not only the age of the child but also the motivation for treatment, how he or she perceives the problem. 

There are different treatment approaches for the correction of anterior dental crossbite which can be used in early mixed dentition period. These include tongue blade therapy [20], reverse stainless steel crowns [21], removable Hawley retainer with anterior Z-springs [16] and bonded resin-composite slopes [10]. The tongue blade therapy is successful only with patient cooperation, and there is no precise control of the amount and direction of force applied. The reverse stainless steel crowns have been shown to be successful but the two main disadvantages of using reverse stainless steel crowns are the unaesthetic appearance of the crown form and the limitations of working with an inclined slope that is already formed. A removable appliance also requires patient cooperation and parental supervision [22].

The Catlan's appliance (Lower Inclined Bite Plane) works on the principle of Newton's third law of motion, the resin slope functions to tip an anterior tooth labially while the mandibular tooth is tipped slightly in the lingual direction [21]. This method is a safe, cost effective, rapid and easy alternative for the treatment of crossbite. It is cost effective because it does not involve the use of fixed orthodontic tooth movement procedures. As it is cemented on the incisors, the treatment outcome does not depend on patient cooperation, does not hamper the growth or cause any discomfort to the patient, and treatment is completed in very few visits to the dentist [22]. The drawbacks of this appliance are difficulty in speech, mastication and risk of anterior open-bite if the appliance is cemented for more than 6 weeks [4]. Therefore, weekly examination of the patient and an accurate decision to remove the appliance in case of prolonged treatment time are critical.

## 4. Conclusion

The above mentioned two cases well describe that Catlan's appliance is an acceptable alternative for correction of anterior dental crossbite instead of complicated fixed orthodontic tooth movements. In both the cases reported here, correction of anterior dental crossbite was observed within three weeks, with no damage to teeth or marginal periodontal tissue. The main emphasis should be placed on the diagnosis and evaluation of the malocclusion with consideration on the facial profile and whether the child is benefited from the treatment at this early stage of development. Further studies are required to evaluate other treatment modalities in comparison with this traditional method of correcting anterior dental crossbite.
